# Supplementary material for: Evaluation of predictive maintenance efficiency with the comparison of machine learning models in machining production process in brake industry
Source: PeerJ Comput Sci. 2025 Jul 16;11:e2999. doi: 10.7717/peerj-cs.2999 (PMC12453749; doi:10.7717/peerj-cs.2999)
Supplement: Supplemental Information 3 [file peerj-cs-11-2999-s003.docx]

# Table 23: Performance Metrics of the MLP models

| param_hidden_layer_sizes | param_activation | param_learning_rate_init | mean_test_accuracy | mean_test_precision | mean_test_recall | mean_test_f1 | rank_test_accuracy |
| --- | --- | --- | --- | --- | --- | --- | --- |
| (50,) | relu | 0.001 | 0.941 | 0.93 | 0.952 | 0.941 | 4 |
| (100,) | relu | 0.01 | 0.945 | 0.934 | 0.954 | 0.944 | 2 |
| (50, 50) | relu | 0.001 | 0.943 | 0.932 | 0.951 | 0.941 | 3 |
| (100,) | tanh | 0.01 | 0.939 | 0.928 | 0.95 | 0.939 | 9 |
| (100,) | relu | 0.001 | 0.942 | 0.931 | 0.951 | 0.941 | 5 |
| (50, 100) | relu | 0.005 | 0.944 | 0.933 | 0.953 | 0.943 | 3 |
| (75,) | tanh | 0.01 | 0.938 | 0.927 | 0.948 | 0.937 | 10 |
| (100, 50) | relu | 0.005 | 0.943 | 0.932 | 0.952 | 0.942 | 3 |
| (50, 100, 50) | relu | 0.001 | 0.94 | 0.929 | 0.949 | 0.939 | 6 |
| (150,) | tanh | 0.01 | 0.946 | 0.935 | 0.956 | 0.945 | 1 |
