# Supplementary material for: Evaluation of predictive maintenance efficiency with the comparison of machine learning models in machining production process in brake industry
Source: PeerJ Comput Sci. 2025 Jul 16;11:e2999. doi: 10.7717/peerj-cs.2999 (PMC12453749; doi:10.7717/peerj-cs.2999)
Supplement: Supplemental Information 4 [file peerj-cs-11-2999-s004.docx]

| **Study (Author, Year)** | **Application Area** | **Data Source & Volume** | **Preprocessing & Feature Engineering** | **Models / Algorithms** | **Validation Protocol** | **Performance Metrics** | **Best Results** | **Original Contribution** | **Limitations & Future Work** |
| --- | --- | --- | --- | --- | --- | --- | --- | --- | --- |
| Abbas et al. (2019) | Lost circulation prediction in oil wells | 1,120 records from 385 wells in southern Iraq (geological and operational parameters) | Categorical encoding; mapminmax & mapstd normalization; 25→18 variable selection with fscaret | ANN (Levenberg–Marquardt, TANSIG/LOGSIG), SVM (Gaussian/RBF, polynomial) | 75–25 train/test; field application | Accuracy, sensitivity, specificity | 91% test accuracy with SVM | Comparison of ANN vs. SVM using field data for lost circulation | Retraining required for new fields/formations |
| Abu-Samah et al. (2015) | Predictive maintenance in semiconductor reactors | Event-based manufacturing defect and maintenance records; 23 predictors | Expert-based critical variable selection | Static Bayesian Network (MDL, Tabu opt.); Dynamic BN proposal | Probability curves and validation of inferred rules | Average accuracy (97.2%) | 97.2% accuracy with BN | Failure probability curves and rule extraction with event-based BN | Lack of real-time integration; multiple failure scenarios not addressed |
| Amihai et al. (2018) | KCI estimation in chemical plant pumps | Hourly/6 hourly wireless vibration & temperature; raw FFT-based KCIs | Noise subtraction; KCI extraction with FFT from raw signal | Random Forest regresyon vs. persistence | Random training/testing; RMSE comparison | RMSE | 10-30% lower RMSE than Persistence | KCI prediction and persistence benchmark with real-world sensor data | Single site data; risk of generalizability |
| Amihai et al. (2020) | Machine health forecast (2 weeks ahead) | 2.5 years, 51 sensor data every 6 hours | Exponential moving average (decay=0.875); entity embeddings; continuous→class label derivation with K-Means | Bidirectional GRU + entity embeddings + fully linked + K-Means clustering | Chronological training/validation/testing; ablation study | Accuracy (overall and class-based) | Overall 87.33% accuracy | GRU+embeddings+clustering architecture and systematic ablation analysis | No deployment/deployment details given |
| Amruthnath & Gupta (2018) | Early fault detection of exhaust fan vibrations | 2048 Hz vibration data every 240 min for 12 days | PCA T² statistic; Elbow & NbClust with k=3 clusters | Hierarchical, K-Means, Fuzzy C-Means, GMM | Lead-time analysis (T² threshold) | Lead-time, endogenous clustering measures | Fault detection before 31 observations with T² | Benchmarkable clustering methodology for unlabeled data | Cluster labels require domain knowledge; no direct classification metrics |
| Aydin & Güldamlıoğlu (2017) | Turbofan engine health prediction (C-MAPSS) | NASA C-MAPSS: 3 settings, 21 sensor time series | Low variance/high correlation sensors discarded; 10-12 sensor selection | LSTM (Spark+Elephas) | Training accuracy & loss graphs; no early-stopping | Training accuracy (~85 %) | ~85% training accuracy | Scaled use of LSTM in big data processing framework | Test/validation metrics not reported |
| Borgi et al. (2017) | Robot manipul. pose error monitoring | PLC 3-phase current time series + Leica Laser Tracker exposure data; 155 profiles | Idle period discarding, motion segmentation | Multiple linear regression | Residual analysis, Predicted vs Observed graph | RMSE=0.0916, MSE=0.008 | MSE=0.008, RMSE=0.0916 | Real-time pose error regression from electric current data | Small data set (155); linear model may miss complex dynamics |
| Krishna & Kannadaguli (2020) | IoT-based CNC condition monitoring | Arduino+accelerometer, 1200 Hz, 1 min ×100 readings | No data augmentation; no PCA/feature selection; no balancing | Single ANN (10 hidden layers, LM training) | 50-50% training/testing | MER (Machine Error Rate) | 4% MER for Fresh, 6% MER for Worn (≈95% accuracy) | Edge/enterprise integration proposal not presented; simple IoT prototype |  |
| Justus & Kanagachidambaresan (2022) | CNC signal processing & simple ML | Vibration, temperature, humidity, part diameter - FFT → frequency components on SBC | Raw FFT; feature selection and equalization not detailed | Linear regression & basic ML methods | Unspecified | Accuracy (%) | ~97.6% accuracy | Rapid prototype based on direct frequency analysis on simple hardware | Lacking metric detail and a wide variety of models |
| Kasiviswanathan et al. (2024) | Compilation of wear monitoring on CNC turning centers | Various literature examples; optical, vibration, acoustic emission, temperature data | Signal processing methods such as Fourier, wavelet, EEMD, HHT were examined | Review of supervised, unsupervised, transfer learning models | – | – | – | Systematic classification of multisensor/fusion methods | No experimental dataset or new model development |
| Çekik & Turan (2025) | Uncertainty management with CNC vibration data | Two-year vibration signals; 50-sample window-overlap segments | Normalize, data augmentation; Rough Set based boundary region generation | RoughLSTM (LSTM+Rough Set decision layer) | 70/15/15 training/val/test; ROC-AUC, FPR/FNR | Accuracy, FPR, FNR, AUC | Accuracy=94.3 %, AUC≈0.95 | Hybridization of Rough Set and LSTM; adaptive decision layer for uncertain instances | Threshold and empirical; no real-time endpoint application |
| Gougam et al. (2024) | CNC tool wear monitoring | 315 cuts × 7 sensor signals (50 kHz) in 3 cutting tool experiments → training:630, test:315 | Time series features; selecting the most “stable” features by Relief + intercept ordering | OSVR; comparison: LR, CNN, CNN-ResNet50, SVR | 3 separate train/test split; RMSE & MAE | RMSE, MAE | RMSE = 8.7; MAE = 7.0 | Scenario-cut automatic feature selection + case-by-case regression with OSVR hybrid | Only 3 cutting tools; requires threshold and scenario generalization |
| Selvaraj & Min (2023) | Ultra-precise CNC power-based diagnostics | 153 time/frequency/double-time feature sets with 32 power parameters, 10 Hz, 15-120 s segments | Segment size optimization, standardization; 153 feature extraction (time/freq/t-freq) | Supervised: DT, Bagging, RF, k-NN, SVM; Unsupervised: Mahalanobis, KDE, Isolation Forest | 10-katlı çapraz doğrulama; 3 split | F1-Score, accuracy | Binary F1 = 0.9971 ± 0.0012; Multiclass F1 = 0.9974 ± 0.0018; Anomaly accuracy = 95 % | Cheap energy meter retrofit + real-time AWS/MQTT pipeline + supervised and unsupervised hybrid learning + container-based end-to-end model development and monitoring | Manual labeling requirement; window size-speed/performance trade-off; automatic updating with reinforcement learning envisioned in the future |
| Tambake et al. (2024) | CNC hobbing cutter izleme | Piezo accelerometer vibration data; 500 samples (100 each case) + 802,000 amplitude values in 15-120 s windows | 13 statistical features (mean, std error, etc.), 2 features with PCA; Decision Tree selection | Decision Tree, Bagged Tree, Logistic Regression | 10-fold cross-validation (400/100 split) | Accuracy, macro F1-score | 100% accuracy (all classifiers) | 100% success with PCA+Decision Tree in five-state multiclass classification with minimal feature set | Five states defined only; future work for model interpretability and real-time endpoint integration |
| Paszkiewicz et al. (2023) | Cutting tool life prediction in milling | Haas VF-1 milling machine; vibration and power consumption time series with 8 accelerometers + 12 current transducers | Raw all sensor channels; no additional attribute selection; GridSearchCV with all variables | SVR (RBF), DecisionTreeRegressor, MLPRegressor (1-3 layers, LBFGS & Adam) | 10-fold CV (GridSearchCV) + 20% test set | R² score | MLP (2/3 layers, LBFGS): R²≈0.962/0.939 | Extensive hyperparameter search and benchmarking of regression models with industrial CNC milling data | Single machine and process only; performance on different machines/generalization unknown |
| Soylemezoglu et al. (2010) | CNC spindle headstock bearing prognostics | 8 axis acceleration + 2 axis temperature sensors | Time/frequency features (BPFI, BPFO, CD frequencies, RMS, kurtosis, temperature); normalization; 11→8 feature selection by Taguchi orthogonal arrays & S/N analysis | Mahalanobis Taguchi System (MD-based clustering & linear RUL estimation) | 1/3 training, 2/3 testing; detection/isolation based on MD thresholds | Detection/isolation accuracy, TTF estimation | 100% detection & isolation; satisfactory TTF estimates | Combination of fault detection, isolation and prognostics in one tool with MTS; process-independent, wireless mote compatible end-to-end prognostic solution | Prognostic error metrics not reported; limited testbed diversity |
