# Supplementary material for: Evaluation of predictive maintenance efficiency with the comparison of machine learning models in machining production process in brake industry
Source: PeerJ Comput Sci. 2025 Jul 16;11:e2999. doi: 10.7717/peerj-cs.2999 (PMC12453749; doi:10.7717/peerj-cs.2999)
Supplement: Supplemental Information 5 [file peerj-cs-11-2999-s005.docx]

# Table 12: Performance Metrics of the Adaboost Model

| param_n_estimators | param_learning_rate | mean_test_accuracy | mean_test_precision | mean_test_recall | mean_test_f1 | rank_test_accuracy |
| --- | --- | --- | --- | --- | --- | --- |
| 200.0 | 1.0 | 0.901662 | 0.856985 | 0.964426 | 0.907508 | 1.0 |
| 100.0 | 1.0 | 0.879128 | 0.834767 | 0.945434 | 0.886484 | 2.0 |
| 50.0 | 1.0 | 0.861355 | 0.817354 | 0.931289 | 0.870063 | 3.0 |
| 200.0 | 0.01 | 0.850697 | 0.791437 | 0.952549 | 0.864505 | 4.0 |
| 200.0 | 0.1 | 0.847147 | 0.790286 | 0.94549 | 0.860839 | 5.0 |
| 50.0 | 0.1 | 0.844773 | 0.789435 | 0.940728 | 0.858359 | 6.0 |
| 100.0 | 0.1 | 0.844773 | 0.790608 | 0.938375 | 0.858085 | 6.0 |
| 50.0 | 10.0 | 0.735792 | 0.701467 | 0.819916 | 0.756009 | 8.0 |
| 100.0 | 10.0 | 0.735792 | 0.701467 | 0.819916 | 0.756009 | 8.0 |
| 200.0 | 10.0 | 0.735792 | 0.701467 | 0.819916 | 0.756009 | 8.0 |
