# Supplementary material for: Evaluation of predictive maintenance efficiency with the comparison of machine learning models in machining production process in brake industry
Source: PeerJ Comput Sci. 2025 Jul 16;11:e2999. doi: 10.7717/peerj-cs.2999 (PMC12453749; doi:10.7717/peerj-cs.2999)
Supplement: Supplemental Information 6 [file peerj-cs-11-2999-s006.docx]

# Table 13: Performance Metrics of the Decision Tree Model

| param_criterion | param_max_depth | param_min_samples_split | param_min_samples_leaf | mean_test_accuracy | mean_test_precision | mean_test_recall | mean_test_f1 | rank_test_accuracy |
| --- | --- | --- | --- | --- | --- | --- | --- | --- |
| entropy | 20 | 10 | 1 | 0.936017 | 0.932504 | 0.9407 | 0.936201 | 1 |
| log_loss | 20 | 10 | 1 | 0.936017 | 0.932504 | 0.9407 | 0.936201 | 1 |
| entropy | 20 | 2 | 1 | 0.932474 | 0.940367 | 0.924146 | 0.931533 | 5 |
| log_loss | 20 | 2 | 1 | 0.932474 | 0.940367 | 0.924146 | 0.931533 | 5 |
| entropy | 20 | 10 | 4 | 0.932467 | 0.932324 | 0.933669 | 0.932507 | 9 |
| log_loss | 20 | 10 | 4 | 0.932467 | 0.932324 | 0.933669 | 0.932507 | 9 |
| log_loss | 10 | 10 | 1 | 0.9301 | 0.915843 | 0.947815 | 0.931391 | 13 |
| entropy | 10 | 10 | 1 | 0.9301 | 0.915843 | 0.947815 | 0.931391 | 13 |
| log_loss | 20 | 2 | 4 | 0.928924 | 0.933829 | 0.924146 | 0.928365 | 15 |
| log_loss | 20 | 5 | 4 | 0.928924 | 0.933829 | 0.924146 | 0.928365 | 15 |
