# Supplementary material for: Evaluation of predictive maintenance efficiency with the comparison of machine learning models in machining production process in brake industry
Source: PeerJ Comput Sci. 2025 Jul 16;11:e2999. doi: 10.7717/peerj-cs.2999 (PMC12453749; doi:10.7717/peerj-cs.2999)
Supplement: Supplemental Information 7 [file peerj-cs-11-2999-s007.docx]

# Performance Metrics of the K-Nearest Neighbors (KNN) Model

| param_n_neighbors | param_weights | param_metric | mean_test_accuracy | mean_test_precision | mean_test_recall | mean_test_f1 | rank_test_accuracy |
| --- | --- | --- | --- | --- | --- | --- | --- |
| 3 | uniform | euclidean | 0.909939 | 0.918637 | 0.900588 | 0.909151 | 1 |
| 3 | uniform | minkowski | 0.909939 | 0.918637 | 0.900588 | 0.909151 | 1 |
| 3 | distance | euclidean | 0.907587 | 0.926863 | 0.886387 | 0.905452 | 3 |
| 3 | distance | minkowski | 0.907587 | 0.926863 | 0.886387 | 0.905452 | 3 |
| 3 | distance | manhattan | 0.90641 | 0.926719 | 0.884034 | 0.904061 | 5 |
| 3 | uniform | manhattan | 0.905206 | 0.917835 | 0.89112 | 0.903803 | 6 |
| 5 | distance | manhattan | 0.902846 | 0.928027 | 0.874538 | 0.899889 | 7 |
| 5 | distance | euclidean | 0.898105 | 0.925095 | 0.867395 | 0.894824 | 8 |
| 5 | distance | minkowski | 0.898105 | 0.925095 | 0.867395 | 0.894824 | 8 |
| 7 | distance | manhattan | 0.896915 | 0.923477 | 0.867423 | 0.893738 | 10 |
