# Supplementary material for: Evaluation of predictive maintenance efficiency with the comparison of machine learning models in machining production process in brake industry
Source: PeerJ Comput Sci. 2025 Jul 16;11:e2999. doi: 10.7717/peerj-cs.2999 (PMC12453749; doi:10.7717/peerj-cs.2999)
Supplement: Supplemental Information 8 [file peerj-cs-11-2999-s008.docx]

# Table 15: Performance Metrics of the Random Forest Model

| param_n_estimators | param_max_depth | param_min_samples_split | param_min_samples_leaf | param_criterion | mean_test_accuracy | mean_test_precision | mean_test_recall | mean_test_f1 | rank_test_accuracy |
| --- | --- | --- | --- | --- | --- | --- | --- | --- | --- |
| 100 | 10 | 5 | 1 | gini | 0.958524 | 0.935297 | 0.98577 | 0.959741 | 1 |
| 50 | 10 | 5 | 1 | gini | 0.958516 | 0.935277 | 0.98577 | 0.959729 | 2 |
| 200 | 20 | 10 | 1 | gini | 0.958516 | 0.935277 | 0.98577 | 0.959729 | 2 |
| 200 | 10 | 10 | 2 | gini | 0.958516 | 0.935295 | 0.98577 | 0.959715 | 2 |
| 100 | 10 | 10 | 1 | log_loss | 0.957333 | 0.933172 | 0.985742 | 0.958585 | 7 |
| 100 | 10 | 10 | 1 | entropy | 0.957333 | 0.933172 | 0.985742 | 0.958585 | 7 |
| 200 | 10 | 2 | 2 | gini | 0.957333 | 0.935127 | 0.983417 | 0.958534 | 11 |
| 200 | 10 | 10 | 1 | log_loss | 0.957326 | 0.935082 | 0.983389 | 0.958521 | 12 |
| 200 | 20 | 10 | 1 | log_loss | 0.957326 | 0.935082 | 0.983389 | 0.958521 | 12 |
| 200 | 10 | 10 | 1 | entropy | 0.957326 | 0.935082 | 0.983389 | 0.958521 | 12 |
