# Supplementary material for: Evaluation of predictive maintenance efficiency with the comparison of machine learning models in machining production process in brake industry
Source: PeerJ Comput Sci. 2025 Jul 16;11:e2999. doi: 10.7717/peerj-cs.2999 (PMC12453749; doi:10.7717/peerj-cs.2999)
Supplement: Supplemental Information 9 [file peerj-cs-11-2999-s009.docx]

## Table 16: Performance Metrics of the Naive Bayes Model

| param_var_smoothing | mean_test_accuracy | mean_test_precision | mean_test_recall | mean_test_f1 | rank_test_accuracy |
| --- | --- | --- | --- | --- | --- |
| 0.1 | 0.707389 | 0.760575 | 0.60423 | 0.672788 | 1.0 |
| 0.0129155 | 0.681354 | 0.74482 | 0.538207 | 0.616811 | 2.0 |
| 0.0002154435 | 0.675437 | 0.765333 | 0.505266 | 0.578289 | 3.0 |
| 2.782559e-05 | 0.674253 | 0.786487 | 0.498207 | 0.56758 | 4.0 |
| 0.001668101 | 0.67307 | 0.733226 | 0.514678 | 0.589324 | 5.0 |
| 3.593814e-06 | 0.671886 | 0.778153 | 0.493501 | 0.55945 | 6.0 |
| 1e-09 | 0.66952 | 0.761487 | 0.488796 | 0.550955 | 7.0 |
| 7.742637e-09 | 0.66952 | 0.761487 | 0.488796 | 0.550955 | 7.0 |
| 5.994843e-08 | 0.66952 | 0.761487 | 0.488796 | 0.550955 | 7.0 |
| 4.641589e-07 | 0.66952 | 0.761487 | 0.488796 | 0.550955 | 7.0 |
