# Supplementary material for: Evaluation of predictive maintenance efficiency with the comparison of machine learning models in machining production process in brake industry
Source: PeerJ Comput Sci. 2025 Jul 16;11:e2999. doi: 10.7717/peerj-cs.2999 (PMC12453749; doi:10.7717/peerj-cs.2999)
Supplement: Supplemental Information 10 [file peerj-cs-11-2999-s010.docx]

# Table 17: Performance Metrics of the LightGBM Model

| param_num_leaves | param_learning_rate | param_n_estimators | param_max_depth | param_min_child_samples | mean_test_accuracy | mean_test_precision | mean_test_recall | mean_test_f1 | rank_test_accuracy |
| --- | --- | --- | --- | --- | --- | --- | --- | --- | --- |
| 10.0 | 0.1 | 50.0 | -1.0 | 10.0 | 0.949049 | 0.922337 | 0.981036 | 0.950705 | 1.0 |
| 31.0 | 0.1 | 50.0 | 5.0 | 20.0 | 0.949042 | 0.924254 | 0.978683 | 0.950586 | 2.0 |
| 31.0 | 0.1 | 100.0 | 3.0 | 10.0 | 0.949042 | 0.920584 | 0.983389 | 0.950805 | 2.0 |
| 10.0 | 0.1 | 50.0 | 5.0 | 10.0 | 0.949042 | 0.922529 | 0.981064 | 0.950757 | 2.0 |
| 10.0 | 0.1 | 100.0 | 3.0 | 10.0 | 0.949042 | 0.920584 | 0.983389 | 0.950805 | 2.0 |
| 20.0 | 0.1 | 100.0 | 3.0 | 10.0 | 0.949042 | 0.920584 | 0.983389 | 0.950805 | 2.0 |
| 20.0 | 0.1 | 50.0 | 5.0 | 20.0 | 0.949042 | 0.924254 | 0.978683 | 0.950586 | 2.0 |
| 10.0 | 0.1 | 50.0 | -1.0 | 20.0 | 0.947866 | 0.924059 | 0.976331 | 0.949377 | 8.0 |
| 10.0 | 0.1 | 50.0 | 5.0 | 20.0 | 0.947866 | 0.924024 | 0.976359 | 0.949394 | 8.0 |
| 20.0 | 0.1 | 50.0 | 5.0 | 10.0 | 0.947852 | 0.922095 | 0.978655 | 0.949483 | 10.0 |
