# Supplementary material for: Evaluation of predictive maintenance efficiency with the comparison of machine learning models in machining production process in brake industry
Source: PeerJ Comput Sci. 2025 Jul 16;11:e2999. doi: 10.7717/peerj-cs.2999 (PMC12453749; doi:10.7717/peerj-cs.2999)
Supplement: Supplemental Information 11 [file peerj-cs-11-2999-s011.docx]

# Table 18: Performance Metrics of the CatBoost Model

| param_iterations | param_learning_rate | param_depth | param_l2_leaf_reg | mean_test_accuracy | mean_test_precision | mean_test_recall | mean_test_f1 | rank_test_accuracy |
| --- | --- | --- | --- | --- | --- | --- | --- | --- |
| 200.0 | 0.1 | 4.0 | 3.0 | 0.954952 | 0.934732 | 0.978655 | 0.956103 | 1.0 |
| 200.0 | 0.2 | 4.0 | 5.0 | 0.951395 | 0.936612 | 0.96916 | 0.952289 | 2.0 |
| 200.0 | 0.1 | 6.0 | 3.0 | 0.951388 | 0.94039 | 0.964426 | 0.952092 | 3.0 |
| 300.0 | 0.1 | 4.0 | 3.0 | 0.951388 | 0.93817 | 0.966779 | 0.952147 | 3.0 |
| 100.0 | 0.1 | 6.0 | 1.0 | 0.950211 | 0.934189 | 0.969216 | 0.951151 | 5.0 |
| 100.0 | 0.2 | 4.0 | 1.0 | 0.950211 | 0.930317 | 0.97395 | 0.951546 | 6.0 |
| 100.0 | 0.1 | 8.0 | 1.0 | 0.950211 | 0.932243 | 0.971597 | 0.951286 | 6.0 |
| 300.0 | 0.1 | 8.0 | 3.0 | 0.950204 | 0.938055 | 0.964426 | 0.950909 | 8.0 |
| 100.0 | 0.2 | 6.0 | 3.0 | 0.949028 | 0.933977 | 0.966891 | 0.95 | 9.0 |
| 200.0 | 0.1 | 4.0 | 5.0 | 0.949028 | 0.93377 | 0.966835 | 0.949976 | 10.0 |
