# Supplementary material for: Evaluation of predictive maintenance efficiency with the comparison of machine learning models in machining production process in brake industry
Source: PeerJ Comput Sci. 2025 Jul 16;11:e2999. doi: 10.7717/peerj-cs.2999 (PMC12453749; doi:10.7717/peerj-cs.2999)
Supplement: Supplemental Information 12 [file peerj-cs-11-2999-s012.docx]

# Table 19: Performance Metrics of the Support Vector Machine (SVM) Model

| Param_C | param_gamma | param_kernel | mean_test_accuracy | mean_test_precision | mean_test_recall | mean_test_f1 | rank_test_accuracy |
| --- | --- | --- | --- | --- | --- | --- | --- |
| 0.1 | Scale | Rbf | 0.899152 | 0.874732 | 0.908655 | 0.896103 | 1.0 |
| 1 | Scale | Rbf | 0.881395 | 0.876612 | 0.880916 | 0.882289 | 2.0 |
| 10 | Scale | Rbf | 0.891388 | 0.84039 | 0.884426 | 0.832092 | 1.0 |
| 0.1 | 0.01 | Linear | 0.831265 | 0.820817 | 0.886779 | 0.872147 | 5.0 |
| 10 | 0.01 | Linear | 0.870211 | 0.864189 | 0.859216 | 0.861151 | 3.0 |
| 100 | 0.001 | Rbf | 0.860211 | 0.830317 | 0.840395 | 0.851546 | 4.0 |
| 1 | 0.001 | Linear | 0.840211 | 0.812243 | 0.891597 | 0.831286 | 6.0 |
| 0.1 | Auto | Linear | 0.820204 | 0.828055 | 0.884426 | 0.850909 | 7.0 |
| 10 | Auto | Rbf | 0.889028 | 0.863977 | 0.886891 | 0.870121 | 1.0 |
| 100 | 0.1 | linear | 0.829028 | 0.860377 | 0.896835 | 0.869976 | 3.0 |
